# Soluble B7-H3 promotes the invasion and metastasis of pancreatic carcinoma cells through the TLR4/NF-κB pathway

**DOI:** 10.1038/srep27528

**Published:** 2016-06-08

**Authors:** Chao Xie, Danqing Liu, Qijun Chen, Chong Yang, Bo Wang, Heshui Wu

**Affiliations:** 1 Pancreatic Disease Institute, Union Hospital, Tongji Medical College, Huazhong University of Science and Technology, Wuhan 430022, Hubei province, People’s Republic of China; 2 Key Laboratory of Ministry of Education for Gastrointestinal Cancer, School of Basic Medical Sciences, Fujian Medical University, Fuzhou 350000, Fujian province, People’s Republic of China; 3 Organ Transplantation Center, Hospital of the University of Electronic Science and Technology of China and Sichuan Provincial People’s Hospital, Chengdu 610072, People’s Republic of China

## Abstract

Many studies have demonstrated a relationship between soluble B7-H3 (sB7-H3) and the poor prognosis of patients with malignant tumors, and increasing evidence has shown a connection between sB7-H3 and NF-κB in tumor progression. In the present study, we demonstrate for the first time that sB7-H3 promotes the invasion and metastasis of pancreatic carcinoma cells through the TLR4/NF-κB pathway. In this study, we observed that sB7-H3 was highly expressed in mB7-H3-positive pancreatic carcinoma (PCa) cells. Exogenous sB7-H3 significantly increased NF-κB activity and promoted the migration and invasion of PCa cells. Further studies proved that sB7-H3 first up-regulated TLR4 expression, then activated NF-κB signaling and finally promoted IL-8 and VEGF expression. In contrast, the silencing of TLR4 using a stable short hairpin RNA significantly decreased the sB7-H3-induced activity of NF-κB and the expression of IL-8 and VEGF in PCa cells. *In vivo* animal experiments further demonstrated that TLR4-knock-down tumor cells displayed a decreased ability to metastasize compared with the control tumor cells after being induced by sB7-H3. Collectively, these results demonstrate that sB7-H3 promotes invasion and metastasis through the TLR4/NF-κB pathway in pancreatic carcinoma cells.

Pancreatic carcinoma (PCa) is a highly invasive and lethal malignant disease. It is the fourth leading cause of cancer deaths in the United States, and the overall 5-year survival rate for this disease from 2004 to 2010 was 7%^1^. Due to the aggressive nature of PCa, more than 80% of patients are already at an advanced stage when diagnosed with pancreatic cancer, present with local invasion or distant metastasis and are not eligible for surgical removal^2^. Even when complete surgical excision can be performed, the overall 5-year survival rate after surgery remains below 20%^3^,^4^.

B7-H3, a newly discovered member of the B7 family, including its soluble form, sB7-H3, was discovered by Zhang *et al*.^5^ and plays a crucial role in the T-cell-mediated immune response^6^. It is widely expressed in many human organs and cells in the human body at the RNA level, whereas its protein expression is relatively limited. In recent decades, research on membrane-bound B7-H3 (mB7-H3), has revealed an abnormally high expression of mB7-H3 in a wide variety of human tumor tissues, which is closely correlated with an unfavorable progression and a poor prognosis for cancer patients, implying that the mB7-H3 expression level can be used as a tumor biomarker^7^,^8^. However, although the mechanism by which B7-H3 affects tumor progression is not clear, some studies have discovered B7-H3-mediated mechanisms of resistance to anti-cancer drugs, such as paclitaxel and gemcitabine, that act partly through the Jak2/Stat3 pathway and involve increased levels of Mcl-1 and survivin proteins^9^,^10^. Other studies have shown that B7-H3 promotes the expression of IL-8, VEGF and matrix metalloproteinases (MMPs) in the tumoral microenvironment^11^,^12^,^13^. However, the exact nature of the interaction between B7-H3 and cytokines is not understood.

NF-κB is a crucial transcription factor that plays a key role in innate and adaptive immunity. Recent research has documented that the constitutive activation of NF-κB is associated with tumorigenesis, invasion and metastasis in human carcinomas^14^,^15^.

A pro-tumorigenic relationship between NF-κB and stat3^16^ has been demonstrated, in which stat3 induces constitutive NF-κB activity in tumors to promote chemoresistance and tumorigenesis. In addition, NF-κB signaling increases the expression of IL-8, VEGF and MMPs in the tumor microenvironment^17^,^18^. Whether a correlation also exists between B7-H3 and NF-κB that facilitates cancer progression requires further investigation. In the present study, we provide evidence that sB7-H3 increases the activity of NF-κB in a TLR4-dependent manner, which promotes PCa cell invasion and metastasis *in vitro* and *in vivo*.

## Results

### B7-H3-positive human pancreatic cells can release sB7-H3

**Figure 1:**
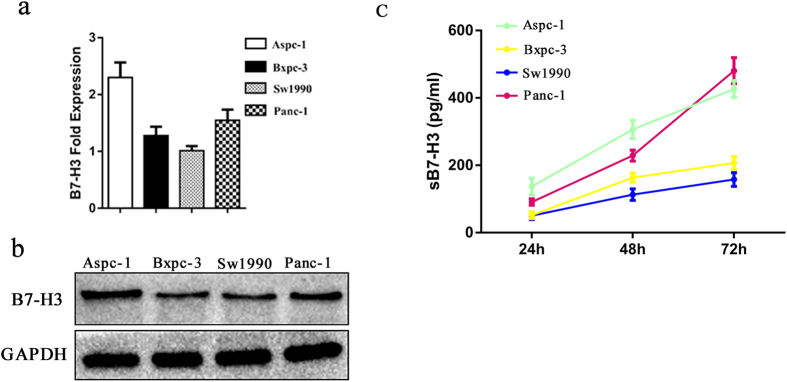
Detection of mB7-H3 and sB7-H3 in PCa cells. (**a,b**) RT-PCR and Western blot analysis for B7-H3 expressed in PCa cells. (**c**) ELISA analysis for sB7-H3 released from PCa cells at various times.

To determine the expression of B7-H3 in pancreatic cells, RT-PCR and Western blot analyses were used to detect B7-H3 in four different types of human pancreatic cancer cells (Fig. 1a,b). To determine whether sB7-H3 was released from pancreatic cells, the cells (1 × 10^5^) were seeded into 6-well plates and cultured for various periods of time, and the cell-free supernatants were tested for the cytokine sB7-H3 (Fig. 1c). Based on the ELISA analysis (Table 1), the levels of sB7-H3 detected in the supernatants from the cultures of Aspc-1, Bxpc-3, Sw1990 and Panc-1 cells varied, and the sB7-H3 levels increased with increasing incubation time. The above findings suggest that B7-H3-positive pancreatic cells can release sB7-H3, which is consistent with the findings of Zhang *et al*. and is an important premise for our study.

### SB7-H3 induces the metastasis and invasion of pancreatic carcinoma cell lines

**Figure 2:**
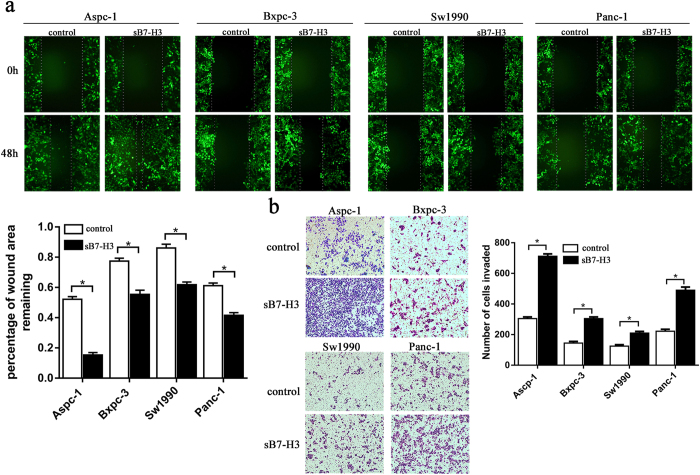
Metastasis and invasion of PCa cell lines in response to sB7-H3. (**a**) The wound healing assay was performed to detect the migration of Aspc-1, Bxpc-3, Sw1990 and Panc-1 cells in response to sB7-H3. The PCa cells were exposed to sB7-H3 (10 μg/ml) for 48 hours after infected by lentivirus with green fluorescent protein (GFP). Representative photographs of wound healing at 0 and 48 hours are shown and the percentage of wound area remaining 48 hours after wounding relative to 0 hour of wounding are quantified (*P < 0.05). (**b**) The Transwell assay was used to determine the invasiveness of PCa cells. The cells were treated with or without sB7-H3 (10 μg/ml) for 48 hours and then incubated in the upper chamber in serum-free medium for 48 hours, while 5% FBS was added into the lower chamber. The cells that invaded through the chamber were quantified (*P < 0.05).

To study whether sB7-H3 could induce the metastasis and invasion of PCa cells, four PCa cell lines were tested using fluorescence-based scratch wound healing and Matrigel invasion assays in the presence or absence of sB7-H3 (10 μg/ml) for 48 hours. The motility and invasion of Aspc-1 cells were increased significantly with sB7-H3 treatment. Similar results were also observed in Sw1990, Bxpc-3 and Panc-1cells, although the effects were not as obvious as those observed in Aspc-1 cells (Fig. 2a,b). Taken together, these results suggest that sB7-H3 can increase PCa cell migration and invasion *in vitro*.

### SB7-H3-induced increase in NF-κB activity may depend on TLR4 in PCa cells

**Figure 3:**
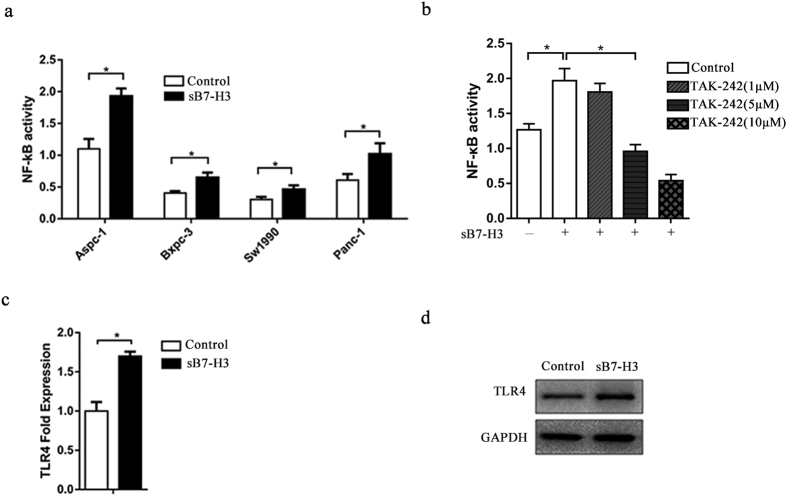
sB7-H3-augmented NF-κB activation may rely on TLR4 in PCa cells. (**a**) A dual-luciferase assay was performed to determine the activity of NF-κB. PCa cells were cotransfected with the NF-κB promoter luciferase and renilla luciferase expression plasmids for 48 hours and further stimulated with or without sB7-H3 (10 μg/ml) for 24 hours (*P < 0.05). (**b**) NF-κB activity was determined using a dual-luciferase reporter assay system in the presence of TAK-242 and sB7-H3. Aspc-1 cells were cotransfected with firefly and Renilla luciferase reporter constructs for 48 hours and further incubated in medium with or without sB7-H3 (10 μg/ml) and TAK-242 (1 μM, 5 μM, or 10 μM) (*P < 0.05). (**c**,**d**) RT-PCR and Western blot analyses were used to evaluate TLR4 expression in the Aspc-1 cells induced with sB7-H3 (*P < 0.05).

To investigate whether sB7-H3 has an impact on the activity of NF-κB in PCa cells, NF-κB activation was induced by sB7-H3 in four PCa cell lines and assessed with a dual-luciferase reporter assay system (Fig. 3a). As shown in the figure, sB7-H3 significantly increased NF-κB transcriptional activity in the PCa cells. The most significant increase was observed in Aspc-1 cells, whereas Sw1990 cells showed the least enhancement. These findings are consistent with the invasion assay and scratch wound healing assay results. Therefore, we chose Aspc-1 cells, the most invasive cell line, for further experimentation. To explore the mechanism between NF-κB activation and sB7-H3, we used TAK-242, a chemical agent that effectively blocks the TLR4 signaling pathway and inhibits TLR4-mediated cellular events. After the addition of TAK-242 to the culture medium in varying concentrations, NF-κB activation induced by sB7-H3 was assessed in Aspc-1 cells. The results show that 1 μM TAK-242 has no obvious impact on NF-κB activation. In contrast, 5 μM TAK-242 significantly decreased NF-κB activation. To further investigate the correlation between sB7-H3 and TLR4, we used RT-PCR and Western blot analyses to detect the expression of TLR4 after induction with sB7-H3 (Fig. 3b,c). As shown by the experimental results, the expression of TLR4 mRNA and protein increased after the treatment of Aspc-1 cells with sB7-H3. These results indicate that the sB7-H3-induced cell invasion and metastasis in PCa cells could be TLR4-dependent.

### SB7-H3 up-regulates the activation of NF-κB through TLR4 in pancreatic cancer cells

**Figure 4:**
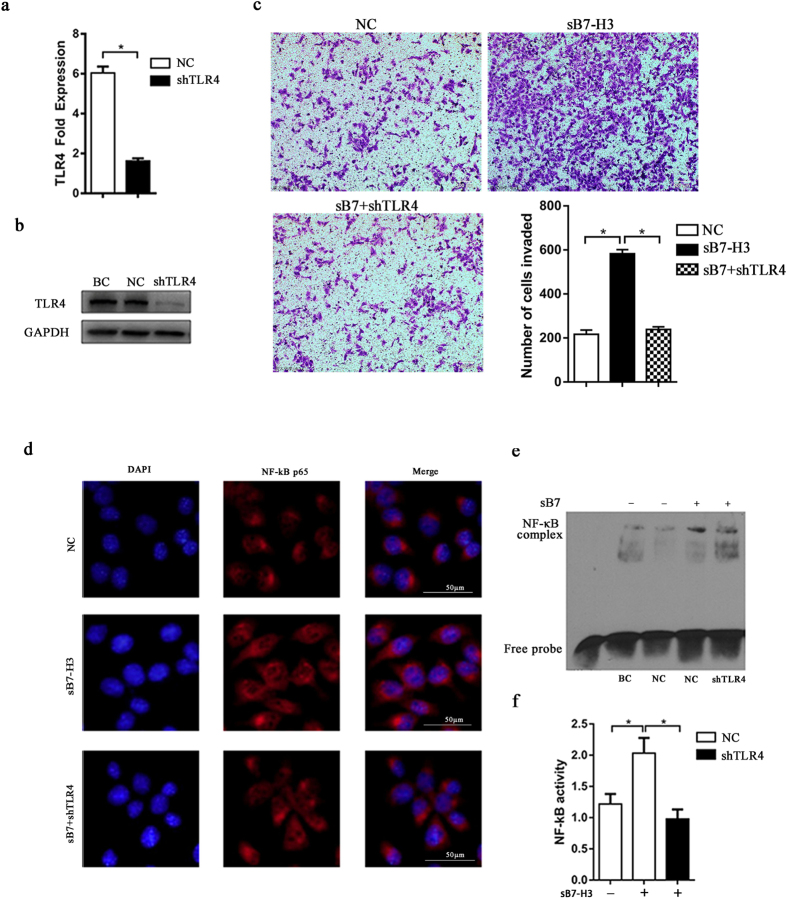
sB7-H3-induced increases in NF-κB activity may depend on TLR4. (**a,b**) RT-PCR and Western blot analyses were used to evaluate the efficiency of TLR4 knock-down in Aspc-1 cells (*P < 0.05). (**c**) The Transwell assay was used to determine the invasiveness of Aspc-1-LV-NC cells and Aspc-1-LV-shTLR4 cells treated with or without sB7-H3 (10 μg/ml) for 48 hours (*P < 0.05). (**d**) Immunofluorescence was used to detect the activation of NF-κB p65 in Aspc-1-LV-NC cells and Aspc-1-LV-shTLR4 cells treated with or without sB7-H3 (10 μg/ml) for 24 hours. In the images, the nuclei are stained blue, and NF-κB p65 is stained red. (**e**) NF-κB binding activity assayed via EMSA in Aspc-1-LV-NC and Aspc-1-LV-shTLR4 cells treated with or without sB7-H3 for 72 hours. (**f** ) NF-κB activity was assayed using a dual-luciferase reporter assay. Aspc-1, Aspc-1-LV-NC and Aspc-1-LV-shTLR4 cells were cotransfected with the NF-κB luciferase and Renilla reporters. Forty-eight hours later, the cells were treated with or withouts B7-H3 for 24 hours and were then analyzed and assayed using a dual-luciferase reporter assay system (*P < 0.05).

To further determine whether TLR4 mediates sB7-H3-induced NF-κB activity, stable TLR4 knock-down lines were established in Aspc-1 cells, and the knock-down efficiency was measured via RT-PCR and Western blot analyses (Fig. 4a,b). TLR4 expression was efficiently knocked down in Aspc-1 cells transfected with shTLR4 compared with its expression in cells transfected with control shRNA. Immunofluorescence was used to observe the activation of NF-κB p65 (Fig. 4c), and there was no nuclear staining of the NF-κB p65 subunit. After the addition of sB7-H3, the nuclear staining of NF-κB p65 was significantly increased in Aspc-1-LV-NC cells. In contrast, the staining of NF-κB p65 was unaffected by sB7-H3 in Aspc-1-LV-shTLR4 cells.

In addition, EMSAs were performed using nuclear protein extracts to test NF-κB binding activity (Fig. 4d). According to the results, NF-κB DNA-binding activity was clearly increased in the presence of sB7-H3 in Aspc-1-LV-NC cells, whereas it was significantly decreased in the TLR4 knock-down Aspc-1 cells. These results are in accordance with the immunofluorescence results. Furthermore, NF-κB activation was assessed with a dual-luciferase reporter system after the NF-κB reporter was transiently transfected into Aspc-1 cells. SB7-H3 was shown to increase NF-κB activity in a TLR4-dependent manner (Fig. 4e), which is consistent with the results of the immunofluorescence and EMSA analyses. The results described above suggest that the expression of TLR4 plays an important role in the constitutive and sB7-H3-mediated NF-κB activity in pancreatic cancer cells.

### SB7-H3 promotes the expression of VEGF and IL-8 through the TLR4/NF-κB pathway

**Figure 5:**
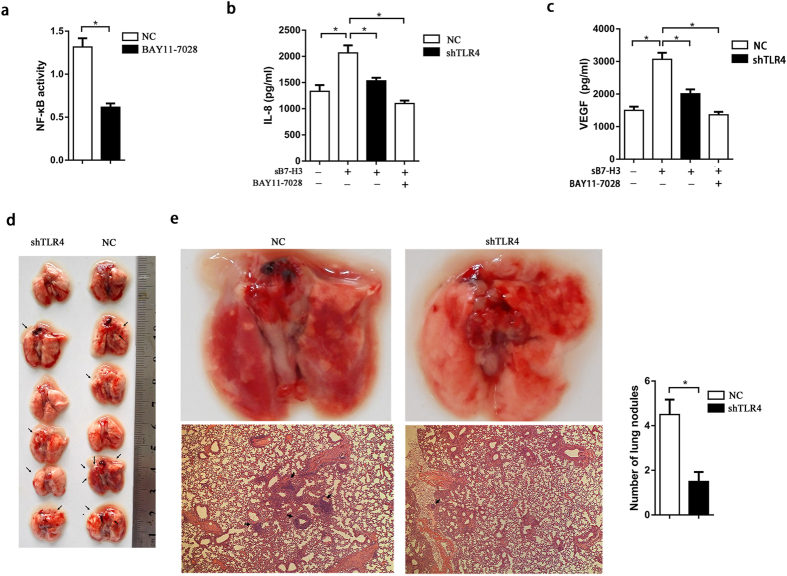
sB7-H3 increased the expression of VEGF and IL-8 through the TLR4/NF-κB signaling pathway. (**a**) Inhibitory effect of BAY 11-7028 on NF-κB activity was assessed with the NF-κB- luciferase reporter vector in Aspc-1-LV-NC cells (*P < 0.05). (**b,c**) The expression levels of IL-8 and VEGF in culture supernatants from Aspc-1 cells under varying conditions after 48 hours were assessed with ELISA kits (*P < 0.05). (**d**) Resected metastatic lung tissue samples from mice injected with Aspc-1-LV-NC and Aspc-1-LV-TLR4 cells induced with sB7-H3. The sites of the tumors are marked with arrows. (**e**) Representative lung tissues from the mice were removed and analyzed with HE staining (100×). The total number of metastatic nodules in the lungs were counted (*P < 0.05).

Constitutive NF-kB activation increases the expression of IL-8 and VEGF, which in turn play important roles in tumor metastasis and the aggressive, biological characteristics of human pancreatic cancer^19^. To determine whether the TLR4/NF-κB signaling pathway is involved in sB7-H3-mediated VEGF and IL-8 induction, Aspc-1, Aspc-1-LV-NC and Aspc-1-LV-shTLR4 cells were exposed to sB7-H3 for 48 h, with BAY 11-7082 being added to the medium to inhibit NF-κB activity in Aspc-1-LV-NC cells at the same time as sB7-H3. First, the inhibition efficiency of BAY 11-7028 on NF-κB activity was assessed using an NF-κB-Luc reporter vector. BAY 11–7082 (10 μM) showed potent inhibitory effects on the activation of NF-κB in Aspc-1-LV-NC cells (Fig. 5a). Next, IL-8 and VEGF protein levels in the culture supernatants from Aspc-1 cells were assessed with ELISA kits. The expression levels of IL-8 and VEGF were dramatically increased after induction with sB7-H3, whereas the protein expression levels were significantly decreased in Aspc-1-LV-shTLR4 cells compared with their expression in Aspc-1-LV-NC cells. The protein levels were also dramatically reduced after inhibition with BAY 11-7028 in Aspc-1-LV-NC cells (Fig. 5b,c). These results strongly suggest the involvement of the TLR4/NF-κB pathway in the secretion of IL-8 and VEGF induced by sB7-H3 in PCa cells. This provides further evidence that sB7-H3 promotes the invasion and metastasis of pancreatic carcinoma cells via an increased expression of IL-8 and VEGF.

To determine whether the inhibition of IL-8 and VEGF secretion via the TLR4/NF-κB pathway can suppress sB7-H3-mediated tumor metastasis in PCa cells, we performed *in vivo* experiments using a mouse model of spontaneous human pancreatic cancer lung metastasis. The Aspc-1-LV-shTLR4 and Aspc-1-LV-NC cells induced with sB7-H3 were resuspended in PBS and injected into the tail veins of mice. Six weeks after the injection, all the mice were sacrificed, and their lungs were removed to analyze the metastatic nodules (Fig. 5d). The lungs of the mice were obtained and stained with HE to determine whether the nodules were metastatic lung cancer. The number of lung metastatic nodules from mice injected with sB7-H3-induced Aspc-1-LV-NC cells was significantly greater than that found in mice injected with sB7-H3-induced Aspc-1-LV-shTLR4 cells (Fig. 5e). These results provide further evidence that sB7-H3 promotes the invasion and metastasis of pancreatic carcinoma cells through the TLR4/NF-κB pathway.

## Discussion

SB7-H3 is a soluble form of B7-H3 that is released by monocytes, activated T cells, DCs and B7-H3-positive tumor cells^5^. In the present study, we confirmed previous results using pancreatic cancer cell lines. To study the relationship between sB7-H3 and NF-κB, we selected four different PCa cell lines (Aspc-1, Bxpc-3, Sw1990, and Panc-1) and carefully assessed the direct effects of sB7-H3 on the invasion and migration of these cells. We demonstrated that sB7-H3 could enhance the invasive and migratory potential of PCa cells through the NF-κB pathway. Further studies suggested that sB7-H3 could activate the NF-κB signaling pathway via a TLR4-dependent mechanism in PCa cells.

NF-κB activity is important for immune system function, whereas inappropriate NF-κB activation can induce an inflammatory reaction and tumorigenesis. Increasing evidence suggests that constitutive NF-κB activity plays a major role in the progression of malignant tumors capable of tissue invasion and metastasis^20^. In this study, we demonstrated that NF-κB activity was up-regulated by sB7-H3 in PCa cells and that increased NF-κB activity may account for the positive correlation between B7-H3-positive tumors and malignant tumorigenesis^21^. In addition, NF-κB regulated numerous gene products, including IL-8 and VEGF, which have been proven to promote tumor invasion and migration by inducing angiogenesis^22^. Previous studies have indicated that B7-H3 is expressed in a high proportion of tumor-related vascular endothelial cells, which is associated with adverse pathological features and poor clinical outcomes^23^,^24^. According to our research, the expression of IL-8 and VEGF was up-regulated through the TLR4/NF-κB pathway in the presence of sB7-H3 in PCa cells, which suggests that sB7-H3 facilitates the formation of nascent blood vessels by increasing the expression of IL-8 and VEGF. Furthermore, our research suggests that sB7-H3 might play an important role in inducing tumor angiogenesis in PCa, which might represent a potential underlying mechanism for the relationship between B7-H3-positive tumor cells and tumor-related vasculature.

NF-κB is constitutively active in pancreatic carcinoma cells, including cells from tissue samples and in cell lines, which leads to increased proliferation and decreased apoptosis. Some of the underlying mechanisms behind constitutive NF-κB activation in cancer have been well described^25^, and crosstalk between NF-κB and stat3 in cancer has been increasingly studied^16^. Growing evidence has shown that constitutive NF-κB activity requires stat3 in tumor cells^26^, including pancreatic carcinoma cells^27^. Considering the results of a previous study showing that mB7-H3 can up-regulate stat3 together with our results suggests that B7-H3 can not only increase stat3 expression but can also activate NF-κB, which could maintain constitutive NF-κB activity in pancreatic tumors. However, whether mB7-H3-induced stat3 expression and sB7-H3-induced NF-κB activation, occur simultaneously or in a step-wise fashion requires further investigation.

TLR4 is over expressed in malignant tumors and tumor-infiltrating immune cells. TLR4 activation may trigger local and systemic inflammation, creating a regenerative tumor microenvironment favoring local recurrence and metastasis^28^. Our work reveals that sB7-H3 up-regulates the expression of TLR4 and that TLR4 signaling is involved in sB7-H3-induced NF-κB activation. To further confirm the sB7-H3-mediated effects, we repeated our experiments *in vitro* in TLR4-deficient PCa cells and *in vivo* in TLR4-deficient mice. The results were consistent with our preliminary findings in PCa cells. These experiments clearly indicate that TLR4/NF-κB signaling is essential for the sB7-H3-promoted progression of pancreatic cancer.

In summary, our study is the first to prove that the sB7-H3-induced invasion and metastasis of PCa cells occurs through the TLR4/NF-κB signaling pathway. Compared with previous reports focusing on the cellular signaling of mB7-H3, our experimental results show that sB7-H3 activates an alternative signal transduction pathway that leads to the malignant progression of pancreatic cancer. In addition, we found that sB7-H3 induces the expression of VEGF and IL-8, providing more evidence for the sB7-H3-induced invasion and metastasis of pancreatic carcinoma cells through the TLR4/NF-κB pathway. Furthermore, the potential angiogenic effects of sB7-H3 may be an early and vital molecular event in tumorigenesis, which could be adapted for use as a biological marker to predict the malignant progression of pancreatic cancer.

## Materials and Methods

### Reagents and antibodies

RPMI 1640 medium, DMEM (Dulbecco’s Modified Eagle Medium), fetal bovine serum (FBS), and a 0.02% ethylenediaminetetraacetic acid (EDTA) solution were obtained from Invitrogen (Gibco BRL, Grand Island, NY, USA). The human B7-H3 antibody, recombinant human B7-H3 protein, human B7-H3 ELISA kit, and the IL-8 and VEGF immunoassay ELISA kits were purchased from R&D Systems (Minneapolis, MN, USA). The PGL4.32 (Luc2p/NF-κB-RE/Hygro) luciferase reporter vector, the PRL-TK vector dual-luciferase reporter assay system and the FuGENE 6 Transfection Reagent were purchased from Promega (Madison, WI, USA). The anti-TLR4 and anti-NF-κB p65 antibodies were obtained from Abcam (Cambridge, UK), the NF-kB inhibitor BAY11-7028 was obtained from Sigma (St. Louis, MO, USA), and the TLR4 inhibitor TAK-242 was purchased from InvivoGen (San Diego, CA, USA).

### Cells and cell cultures

The human pancreatic cancer (PCa) cell lines Aspc-1, Bxpc-3, Sw1990, and Panc-1 were purchased from the Chinese Academy of Science Cell Bank. The Panc-1 cells were cultured in DMEM, and the other cells were cultured in RPMI 1640 medium. All media were supplemented with 10% FBS. Cells were maintained at 37 °C and 5% CO_2_ in a humidified atmosphere. For the recombinant human B7-H3 treatment, the cells were treated with 10 μg/ml for 24, 48, or 72 hours. For TLR4 signal inhibition, varying concentrations of TAK-242(1 μM, 5 μM, or 10 μM) were added to the cell culture medium for 24 hours. For the NF-κB inhibition, BAY11-7028 (10 μM) was added to the cell culture medium at the same time as sB7-H3 for 48 hours.

To further confirm our results, stable TLR4 knock-down cell lines were established. Lentiviral vectors encoding the green fluorescent protein (GFP) sequence containing shRNA specific to TLR4 (LV-shTLR4) and a non-targeted control shRNA (LV-NC) were constructed by Shanghai Genechem Co. (Shanghai, China), and the lentiviruses were titered to 5 × 10^9^ TU/ml. Stable shRNA-silenced lines were established in Aspc-1cells using lentiviral vectors according to the manufacturer’s instructions. Briefly, LV-shTLR4 and LV-NC, each at a multiplicity of infection (MOI) of 50, were transfected into cells (2 × 10^4^) in 12-well plates with 5 μg/ml of Polybrene. The sequence of the TLR4 shRNA used in this study is as follows: 5′-AACCCGGAGGCCATTATGCTA-3′.

### Scratch wound healing and Transwell assays

For the scratch wound healing assay, cells (50 × 10^4^) were seeded into 6-well plates and incubated overnight. The monolayer was then scraped with a sterile 10 μl pipet tip when the cells reached 90% confluence, and photographs of three regions per scratch were taken at 0 and 48 hours under a phase-contrast microscope. The experiments were carried out in triplicate and representative images were analyzed using ImageJ software. For the Transwell assay, approximately 5 × 10^4^ cells in 200 μl of serum-free DMEM medium were seeded into the upper chambers containing pores coated with 200 μg/ml of Matrigel (BD, USA), and 500 μl of DMEM containing 10% FBS was added into the lower chamber as a chemoattractant. After incubation at 37 °C in 5% CO_2_ atmospheric conditions for 48 hours, the cells were rinsed with PBS, fixed in 4% formaldehyde for 30 minutes and stained with crystal violet dye. The cells on the upper surface of the chambers were removed, and the number of cells that invaded through the pores was counted in 5 random fields at 200x magnification under a microscope (Olympus, Tokyo). The data were then statistically analyzed.

### Transient transfection and luciferase assays

Cells (1 × 10^5^) were plated in 6-well plates overnight prior to transfection. Then, the cells were co-transfected with 2 μg of the NF-κB-luc reporter construct and 0.1 μg of the renilla luciferase-expression plasmid using 6 μl of FuGENE 6. At 48-hours post-transfection, the cells were treated with sB7-H3, TAK-242 or BAY11-7028 and then lysed, and the activities of firefly and renilla luciferases were assessed using a dual-luciferase reporter assay system. All the experiments were performed in triplicate.

### Immunofluorescence

Approximately 2 × 10^4^ cells were seeded onto a 24-well dish. After treatment, the cells were washed three times with PBS, fixed in 4% cold paraformaldehyde for 20 minutes, and incubated in TritonX-100 in PBS for 10 minutes. Next, the cells were blocked in 10% goat serum and then incubated overnight at 4 °C with a rabbit anti-rabbit NF-κB p65 antibody. After being washed with PBS, the cells were incubated with a secondary antibody for 2 hours at room temperature in the dark. Finally, the cells were photographed under a fluorescence microscope after being stained with DAPI/PI for 5 minutes.

### RT-PCR

RT-PCR was used to measure the B7-H3 mRNA levels in four pancreatic cancer cell lines and the TLR4 mRNA levels after transfection with shTLR4. Total RNA was extracted using TRIzol reagent, first strand cDNA was synthesized from 0.5 μg of RNA using a cDNA Reverse Transcription kit, and real-time PCR analysis was carried out with a PCR kit according to the manufacturer’s protocols. The two kits were purchased from Takara Bio Inc. (Shigo, Japan). The mRNA levels of the target gene were normalized to control values of GAPDH. The sequence for the human B7-H3, TLR4 and GAPDH primers used in real-time PCR were as follows: B7-H3 forward primer: 5′-GTGGGGCTGTCTGTCTGTCTCAT-3′, reverse primer: 5′-GCTGTCAGAGTGTTTCAGAGGCT-3′; TLR4 forward primer: 5′-CTGCAATGGATCAAGGACCA-3′, reverse primer: 5′-TTATCTGAAGGTGTTGCACATTCC-3′; GAPDH forward primer: 5′-GTCACCAGGGCTGCTTTTAACTC-3′, reverse primer: 5′-CAGCATCGCCCCACTTGATTTTG-3′.

### Western blotting

Cells (1 × 10^4^) were seeded into 6-well plates in 2 ml of medium and cultured overnight. Then, the cells received various treatments for 48 or 72 hours. After the treatment, the cells were washed three times with PBS and lysed with 160 μl of lysis buffer containing 1% protease and phosphatase inhibitors and PMSF for 30 minutes on ice. Then, the cell lysates were centrifuged at 12,000 × *g* for 20 minutes at 4 °C to remove un-dissolved impurities, and the supernatants were collected. In addition, nuclear protein extracts were prepared using the Thermo scientific NE-PEP kit (Thermo Pierce, USA) according to manufacturer’s protocol. Briefly, after trypsinization and centrifugation, the cells were washed with PBS and suspended in a cytoplasmic extraction reagent. Then, after centrifugation for 5 minutes at 16,000 × *g*, the supernatant was removed and resuspended in nuclear extraction reagent. The nuclear extracts were obtained after another centrifugation for 10 minutes, and then the extracts were kept frozen at −80 °C until analyzed. Protein content was determined with a protein assay kit (Pierce Biotechnology). Next, 10–30 μg of protein was separated using SDS-PAGE (10%) and transferred onto PVDF membranes(Pierce Biotechnology), and then, the PVDF membranes were blocked in 5% not-fat milk for 1 hour at room temperature and incubated with a primary antibody against TLR4 diluted in a primary antibody dilution buffer (Beyotime Suzhou China) overnight at 4 °C. The membranes were then washed three times with 1xTBST and incubated with a secondary antibody for 1 hour. Finally, the membranes were treated with ECL detection reagents and exposed to X-ray film to detect the protein bands.

### EMSA

To provide evidence that NF-κB was constitutively activated in the PCa cells, an EMSA was performed with nuclear extracts from cells treated with B7-H3 using the LightShift Chemiluminescent EMSA kit (Thermo Pierce USA) following the manufacturer’s instructions. Briefly, double-stranded oligonucleotide NF-κB probes with a biotin 3′-end-labeling sequence (5′-AGTTGAGGGGACTTTCCCAGGC-3′)were added to the reaction mixture. Each binding reaction mixture consisted of 10x binding buffer (2 μl), 20 fmol biotin end-labeled target DNA (2 μl), nuclear extract (5 μg), poly(dI:dc) (1 μl), and ddH_2_O (13 μl). The binding reaction was carried out at room temperature for 20 minutes, and then, 2 μl of the 10x binding buffer was added and samples were electrophoresed through polyacrylamide gels and transferred to a nylon membrane for 30 minutes at 380 mA. Next, DNAs were cross-linked to the membrane under anultraviolet lamp for 10 minutes. The biotin-labeled DNA was exposed to X-ray film after being incubated in the substrate working solution for 5 minutes.

### ELISA

To measure the levels of cytokines (sB7-H3, IL-8, and VEGF) in the supernatants, we used three ELISA kits from R&D Systems according to the manufacturer’s instructions. Briefly, cell-free supernatants were immediately centrifuged and then transferred to a 96-well plate. After being incubated for 2 h at room temperature, every well was washed four times, and 200 μl of conjugate was added and incubated for another 2 hours at room temperature. Next, 200 μl of substrate solution was added into each well and incubated for 20–30 minutes in the dark. Then, the color was developed by adding a stop solution, and the absorbance was measured at 450/540 nm.

### Animals

8- to 10-wk-old C_3_H/HeJ mice were purchased from the Animal Experimental Center of Wuhan University. The mice were bred in aseptic specific-pathogen-free (SPF) conditions with a constant humidity and temperature (25–28 °C) in the University Biological Services Unit. All animal experiments were performed according to the Guidelines set forth by the Chinese National Institutes of Health and followed the protocols approved by the Ethical Committee on Animal Experiments of the Huazhong University of Science and Technology, Wuhan, China. After stimulation with sB7-H3 for 48 hours *in vitro*, Aspc-1 (LV-shTLR4 or control) cells (1 × 10^6^) suspended in 50 μl of PBS were injected into the caudal veins of mice (2 groups, 6 mice per group). Six weeks after the injection of the cells, all the mice were sacrificed, and their lungs were harvested and stained with HE to determine the number of metastatic lung nodules.

### Statistical analysis

SPSS software version 20.0 (SPSS, Chicago, IL, USA) was used for the statistical analyses. All the data are presented as the mean ± the standard error of the mean (SEM) and analyzed using Student’s t-tests or one-way ANOVAs followed the Student-Newman-Keuls (SNK) tests. Values of p that were less than 0.05 were considered to be statistically significant.

## Additional Information

**How to cite this article**: Xie, C. *et al*. Soluble B7-H3 promotes the invasion and metastasis of pancreatic carcinoma cells through the TLR4/ NF-κB pathway. *Sci. Rep*. **6**, 27528; doi: 10.1038/srep27528 (2016).

## Figures and Tables

**Table 1:** Cellular supernatant sB7-H3 content in four PCa cells (pg/ml).

	24 h		48 h		72 h	
	mean ± SE	range	mean ± SE	range	mean ± SE	range
Aspc-1	137.41 ± 14.47	(75.14–199.68)	306.83 ± 15.63	(239.58–374.07)	426.45 ± 14.66	(363.35–489.54)
Bxpc-3	52.47 ± 5.78	(27.58–77.36)	163.72 ± 7.83	(130.03–197.41)	207.07 ± 11.19	(158.93–255.33)
Sw1990	50.92 ± 6.62	(22.42–79.42)	113.46 ± 9.81	(71.24–155.69)	158.15 ± 11.69	(107.85–208.46)
Panc-1	91.32 ± 5.56	(67.42–115.23)	229.05 ± 9.32	(188.95–269.15)	480.99 ± 22.34	(384.87–577.11)

## References

[b1] Siegel R. L., Miller K. D. & Jemal A. Cancer statistics, 2015. CA Cancer J Clin 65, 5–29, doi: 10.3322/caac.21254 (2015). 25559415

[b2] Campen C. J., Dragovich T. & Baker A. F. Management strategies in pancreatic cancer. Am J Health Syst Pharm 68, 573–584, doi: 10.2146/ajhp100254 (2011). 21411798

[b3] Riediger H. . The lymph node ratio is the strongest prognostic factor after resection of pancreatic cancer. J Gastrointest Surg 13, 1337–1344, doi: 10.1007/s11605-009-0919-2 (2009). 19418101

[b4] Matsuoka L., Selby R. & Genyk Y. The surgical management of pancreatic cancer. Gastroenterol Clin North Am 41, 211–221, doi: 10.1016/j.gtc.2011.12.015 (2012). 22341259

[b5] Zhang G. . Soluble CD276 (B7-H3) is released from monocytes, dendritic cells and activated T cells and is detectable in normal human serum. Immunology 123, 538–546, doi: 10.1111/j.1365-2567.2007.02723.x (2008). 18194267 PMC2433324

[b6] Chapoval1 A. I. & N. J. B7-H3: A costimulatory molecule for T cell activation and IFN-γ production. Nat Immunology 2, 269–274 (2001). 11224528 10.1038/85339

[b7] Roth T. J. . B7-H3 ligand expression by prostate cancer: a novel marker of prognosis and potential target for therapy. Cancer Res 67, 7893–7900, doi: 10.1158/0008-5472.CAN-07-1068 (2007). 17686830

[b8] Arigami T. . B7-H3 expression in gastric cancer: a novel molecular blood marker for detecting circulating tumor cells. Cancer Sci 102, 1019–1024, doi: 10.1111/j.1349-7006.2011.01877.x (2011). 21251161

[b9] Liu H. . B7-H3 silencing increases paclitaxel sensitivity by abrogating Jak2/Stat3 phosphorylation. Mol Cancer Ther 10, 960–971, doi: 10.1158/1535-7163.mct-11-0072 (2011). 21518725 PMC3253760

[b10] Zhao X. . Silencing of B7-H3 increases gemcitabine sensitivity by promoting apoptosis in pancreatic carcinoma. Oncol Lett 5, 805–812, doi: 10.3892/ol.2013.1118 (2013). 23426281 PMC3576185

[b11] Tekle C. . B7-H3 contributes to the metastatic capacity of melanoma cells by modulation of known metastasis-associated genes. Int J Cancer 130, 2282–2290, doi: 10.1002/ijc.26238 (2012). 21671471

[b12] Xu L., Ding X., Tan H. & Qian J. Correlation between B7-H3 expression and matrix metalloproteinases 2 expression in pancreatic cancer. Cancer Cell Int 13, 81, doi: 10.1186/1475-2867-13-81 (2013). 23947693 PMC3751640

[b13] Kang F. B., Wang L., Li D., Zhang Y. G. & Sun D. X. Hepatocellular carcinomas promote tumor-associated macrophage M2-polarization via increased B7-H3 expression. Oncol Rep 33, 274–282, doi: 10.3892/or.2014.3587 (2015). 25370943

[b14] Maier H. J. . NF-kappaB promotes epithelial-mesenchymal transition, migration and invasion of pancreatic carcinoma cells. Cancer Lett 295, 214–228, doi: 10.1016/j.canlet.2010.03.003 (2010). 20350779

[b15] Huang, Suyun, P., C. A., Uehara, Hisanori, Bucana1, Corazon, D. and Fidler, Isaiah, J. Blockade of NF-kB activity in human prostate cancer cells is associatedwith suppression of angiogenesis, invasion, and metastasis. oncogene 20, 4188–4197 (2001). 11464285 10.1038/sj.onc.1204535

[b16] Grivennikov S. I. & Karin M. Dangerous liaisons: STAT3 and NF-kappaB collaboration and crosstalk in cancer. Cytokine Growth Factor Rev 21, 11–19, doi: 10.1016/j.cytogfr.2009.11.005 (2010). 20018552 PMC2834864

[b17] Bilandzic M. . Betaglycan blocks metastatic behaviors in human granulosa cell tumors by suppressing NFκB-mediated induction of MMP2. Cancer Letters 354, 107–114, doi: 10.1016/j.canlet.2014.07.039 (2014). 25128652

[b18] Karin M. Nuclear factor-kappaB in cancer development and progression. Nature 441, 431–436, doi: 10.1038/nature04870 (2006). 16724054

[b19] Shuichi Fujioka G. M. S., Schmidt Christian . Function of Nuclear Factor KB in Pancreatic Cancer Metastasis. Clinical cancer research 9, 346–354 (2003). 12538487

[b20] Prasad R. & Katiyar S. K. Grape seed proanthocyanidins inhibit migration potential of pancreatic cancer cells by promoting mesenchymal-to-epithelial transition and targeting NF-kappaB. Cancer Lett 334, 118–126, doi: 10.1016/j.canlet.2012.08.003 (2013). 22902508

[b21] Sun T. W., Gao Q., Qiu S. J., Zhou J. & Wang X. Y. B7-H3 is expressed in human hepatocellular carcinoma and is associated with tumor aggressiveness and postoperative recurrence. Cancer Immunol Immunother 61, 2171–2182, doi: 10.1007/s00262-012-1278-5 (2012). 22729558 PMC11029627

[b22] Ferrara N. VEGF and the quest for tumour angiogenesis factors. Nat Rev Cancer 2, 795–803, doi: 10.1038/nrc909 (2002). 12360282

[b23] Zang X. . Tumor associated endothelial expression of B7-H3 predicts survival in ovarian carcinomas. Mod Pathol 23, 1104–1112, doi: 10.1038/modpathol.2010.95 (2010). 20495537 PMC2976590

[b24] Crispen P. L. . Tumor cell and tumor vasculature expression of B7-H3 predict survival in clear cell renal cell carcinoma. Clin Cancer Res 14, 5150–5157, doi: 10.1158/1078-0432.CCR-08-0536 (2008). 18694993 PMC2789387

[b25] Aggarwal B. B. & Sung B. NF-kappaB in cancer: a matter of life and death. Cancer Discov 1, 469–471, doi: 10.1158/2159-8290.cd-11-0260 (2011). 22586649 PMC3392037

[b26] Lee H. . Persistently activated Stat3 maintains constitutive NF-kappaB activity in tumors. Cancer Cell 15, 283–293, doi: 10.1016/j.ccr.2009.02.015 (2009). 19345327 PMC2777654

[b27] Gong J. . Combined targeting of STAT3/NF-kappaB/COX-2/EP4 for effective management of pancreatic cancer. Clin Cancer Res 20, 1259–1273, doi: 10.1158/1078-0432.CCR-13-1664 (2014). 24520096 PMC3969421

[b28] Oblak A. & Jerala R. Toll-like receptor 4 activation in cancer progression and therapy. Clin Dev Immunol 2011, 609579, doi: 10.1155/2011/609579 (2011). 22110526 PMC3216292

